# Ultrasound-guided diagnosis on a parapharyngeal mass

**DOI:** 10.1007/s40477-024-00882-z

**Published:** 2024-03-29

**Authors:** Frisone Alessio, Antonio Scarano, Giulia Valentini, Andrea Boccatonda, Lorenzo Andreetto, Susanna Vicari

**Affiliations:** 1 Department of Innovative Technology in Medicine and Dentistry, University of Chieti-Pescara, Chieti, Italy; 2 Department of Medical, Oral and Biotechnological Sciences, University G. d’Annunzio, 66100 Chieti, Italy; 3 Internal Medicine, Bentivoglio Hospital, AUSL Bologna, Via Marconi 35, Bentivoglio, 40010 Bologna, Italy; 4 Internal Medicine, IRCCS Azienda Ospedaliero-Universitaria Policlinico di Sant’Orsola, Bologna, Italy

**Keywords:** Ultrasound, Cancer, Biopsy, Sarcomatoid, Neck

## Abstract

Masses in the parapharyngeal area are rare and often due to infectious phenomena arising from the oral cavity or pharynx which lead to abscess formation. Less frequently, the lesion can be neoplastic. Tumours of the parapharyngeal space are rare, accounting for less than 1% of all head and neck neoplasms. We report the case of a patient who came to our observation for mandibular pain. Multiparametric diagnostic imaging was done thus showing a parapharyngeal mass. An ultrasound guided biopsy was performed by a transcutaneous route with a high median approach at neck level, to characterize the mass in the right tonsillar region. The histological examination reported the final histological diagnosis of sarcomatoid carcinoma.

## Introduction

Parapharyngeal space (PPS) tumors are rare and represent approximately 0.5–1% of head and neck neoplasms only. Salivary gland tumors and neurogenic tumors are the most common PPS neoplasms [1]. The preoperative diagnosis of PPS tumors is difficult due to their low incidence, anatomical complexity, and histological heterogeneity [2]. Computed tomography (CT) and/or magnetic resonance imaging (MRI) are mostly employed. MRIs generally provide more specific findings, while CT data are quite limited [3, 4]. The tumor locations in the prestyloid or retrostyloid areas as observed by imaging methods may enhance the diagnostic accuracy of presumptive diagnosis [5]. Relevant knowledge and experience are required for radiologists to accurately provide a specific diagnosis based on imaging features of these rare tumors. Biopsy, often by fine-needle aspiration (FNA), is another clinical method in addition to CT and MRI to establish a preoperative diagnosis, especially when performed under image guidance [6]. In this context, ultrasound method seems to be the best method to guide the biopsy procedure, as is well established in some areas such as lymph node biopsies and other superficial masses and structures [7–9].

Here we report the case of a patient who came to our observation for mandibular pain. Multiparametric diagnostic imaging was done thus showing a PPS mass.

## Case report

A 58-year-old woman referred to the emergency room reporting pain in the right hemimandible for 50 days. Patient reported no allergies and no relevant disease except hypothyroidism on treatment. Before going to the emergency room, patient underwent a dentist evaluation, just hypothesizing the odontogenic origin of the pain, and an extraction of the VIII tooth was performed, included in the lower right arch. Since then, pain to the right hemimandible got worse, extending to the right ear with sensation of muffling, hyposensitivity of the right hemilingual and hemipalate and slight deficit of the right facial nerve. Patient did not report odynophagia. General practitioner prescribed antibiotic therapy with amoxicillin/clavulanic acid, without any benefit.

Upon arrival to the emergency room, patient was prostrated with pain, he had no fever, and physical examination demonstrated right hemiface swelling with palpable right later cervical lymphadenopathy. Pain-relief and antibiotic therapy was early prescribed, following infectious disease consultation, to ascertain the nature of the parapharyngeal formation.

Blood tests showed: white blood cells: 11.3 × 10^−9^/L (3.6–10.5); hemoglobin: 13.1 g/dL (12–15.6); PLT: 382 × 10^−9^/L (160–370); creatinine: 0.58 mg/dL (0.5–1.2); sodium: 138 mmol/L (136–145); potassium: 4.0 mmol/L (3.5–5.3); calcium: 9.3 mg/dL (8.6–10.5); AST: 32 U/L (0–35); ALT: 21 U/L (0–35); C-reactive protein: 0.34 mg/dL (0.0–0.5); procalcitonin: < 0.1 (< 0.5); TSH: 1.95 μU/mL (0.25–4.5); CEA: 1.3 (0–5); CA 19.9: 31.7 (0–37).

Otorhinolaryngology consultation was performed, and otoscopy showed right catarrhal otitis and normal left tympanic membrane. In oropharingoscopy, the medialization of the right tonsil could be appreciated with swelling of the ipsilateral hemipalate, but without a clear abscess collection. At laryngeal fibroscopy, glottic plan was normal for morphology and mobility.

**Fig. 1:**
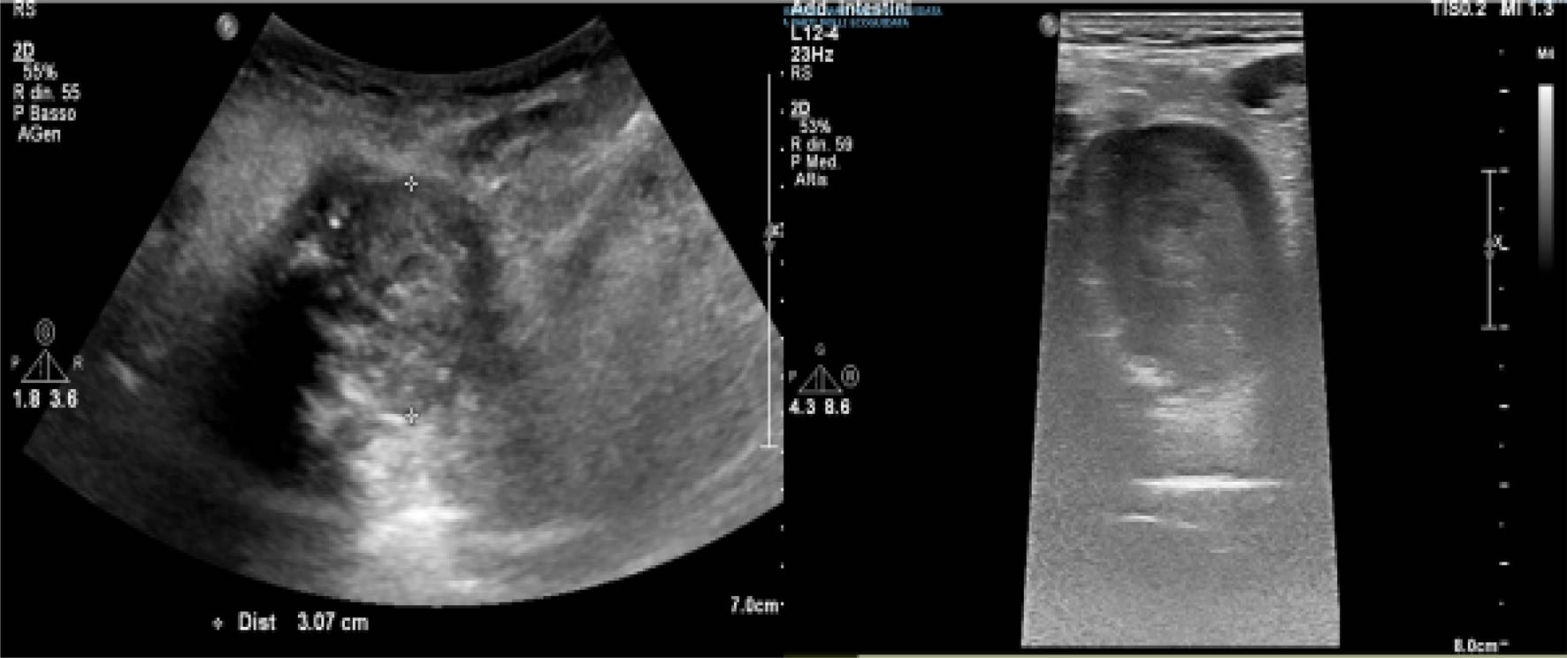
B-mode ultrasound with convex and linear probe in the upper midline of the neck. On the right-side of the pharynx, an oval formation with a non-homogeneous echostructure measuring approximately 30 mm is visible

**Fig. 2:**
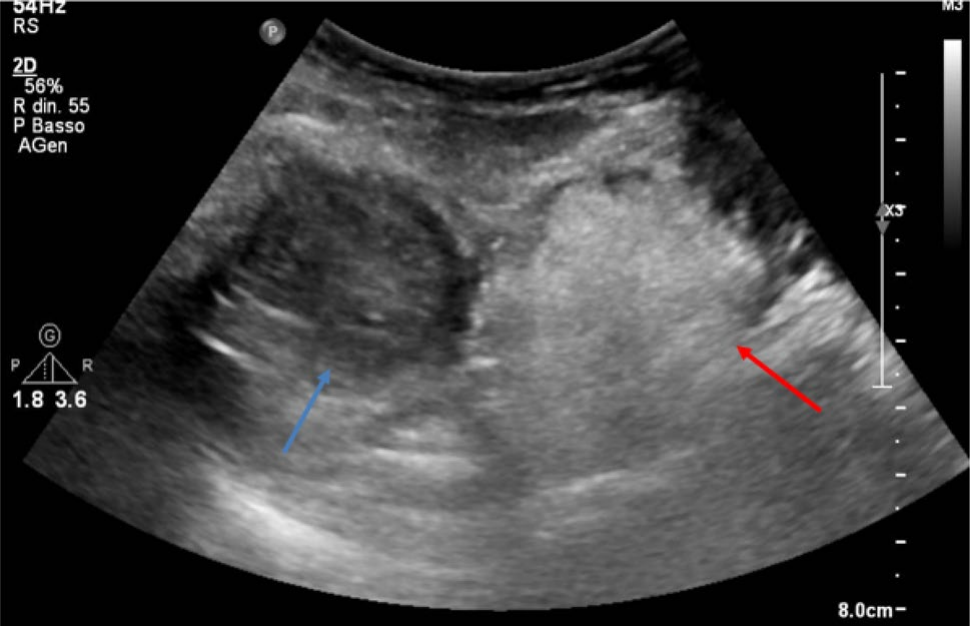
B-mode image with convex probe: detail showing the mass with a non-homogeneous hypoechoic echostructure (blue arrow) close to the tongue (red arrow)

Ultrasound examination of the neck detected a large hypoechoic mass (about 30 mm) near the tongue on the right-side of the pharynx, and some pathological lymph nodes in the submandibular and lateral cervical areas; there were no alterations of the thyroid or the submandibular and parotid glands (Figs. 1 and 2).

**Fig. 3:**
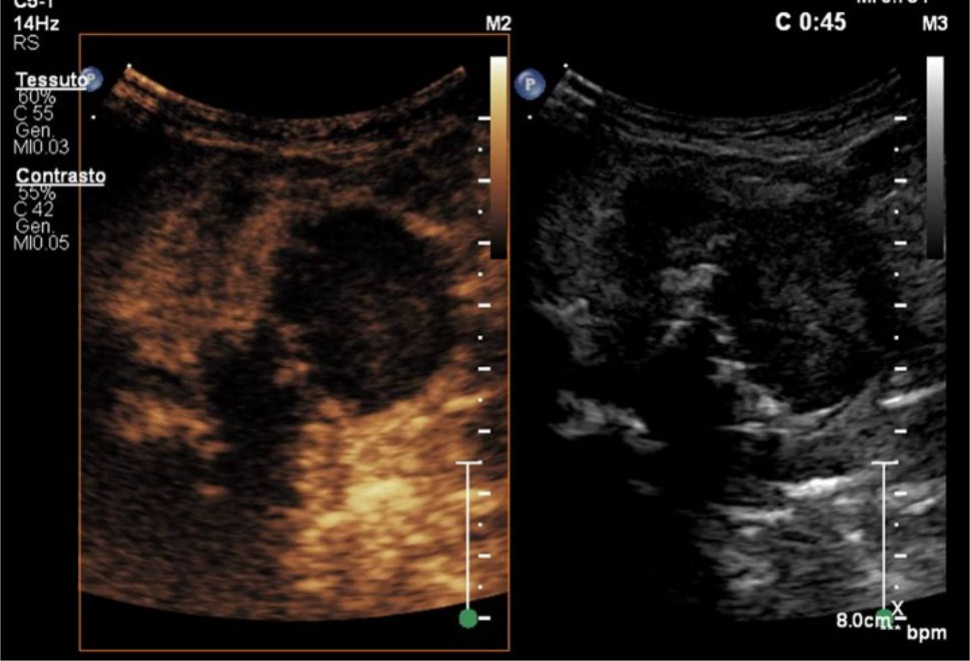
CEUS examination showed lesion periphery hyper-enhancement. The internal portion was not perfused

Furthermore, a contrast-enhanced ultrasound (CEUS) examination was performed (Sonovue™ 2.5 ml) which highlighted a persistent ring-shaped hyperenhancement of the lesion while the rest of the lesion did not receive contrast medium (not perfused area) (Fig. 3).

**Fig. 4:**
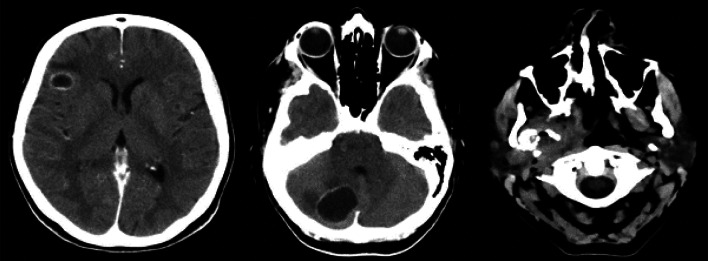
CT scan of the brain and maxillo-facial. The presence of a space-occupying lesion in the right pharyngeal space was confirmed; two lesions were visible at the cerebellar and right frontal level; they presented peripheral enhancement, thus inducing a differential diagnosis between infectious/abscess and neoplastic lesions

A subsequent CT scan confirmed the presence of a voluminous expansive mass (35 × 38 mm diameters) in the right tonsillar region, with a central portion unevenly hypodense, thick, and ragged walls with uneven contrast-enhancement and multiple calcifications. This mass determined a clear compressive-dislocation effect on the adjacent trachea and could not be dissociated from parotid gland and adjacent muscle budles. Multiple adenopathies in the submandibular, laterocervical and supra- and subclavicular area on the right side were detected (Fig. 4).

Brain CT scan demonstrated two rounded and hypodense lesions, one in the right frontal area (14 × 8 mm) and one in the right cerebellar area (19 × 19 mm), with peripheral contrast-enhancement. Such formations demonstrated moderate perilesional edema, without significant compressive phenomena on the nearby structures. Ventricular system was in place and with regular morphology; subarachnoid spaces of the convexity and the base were normal.

**Fig. 5:**
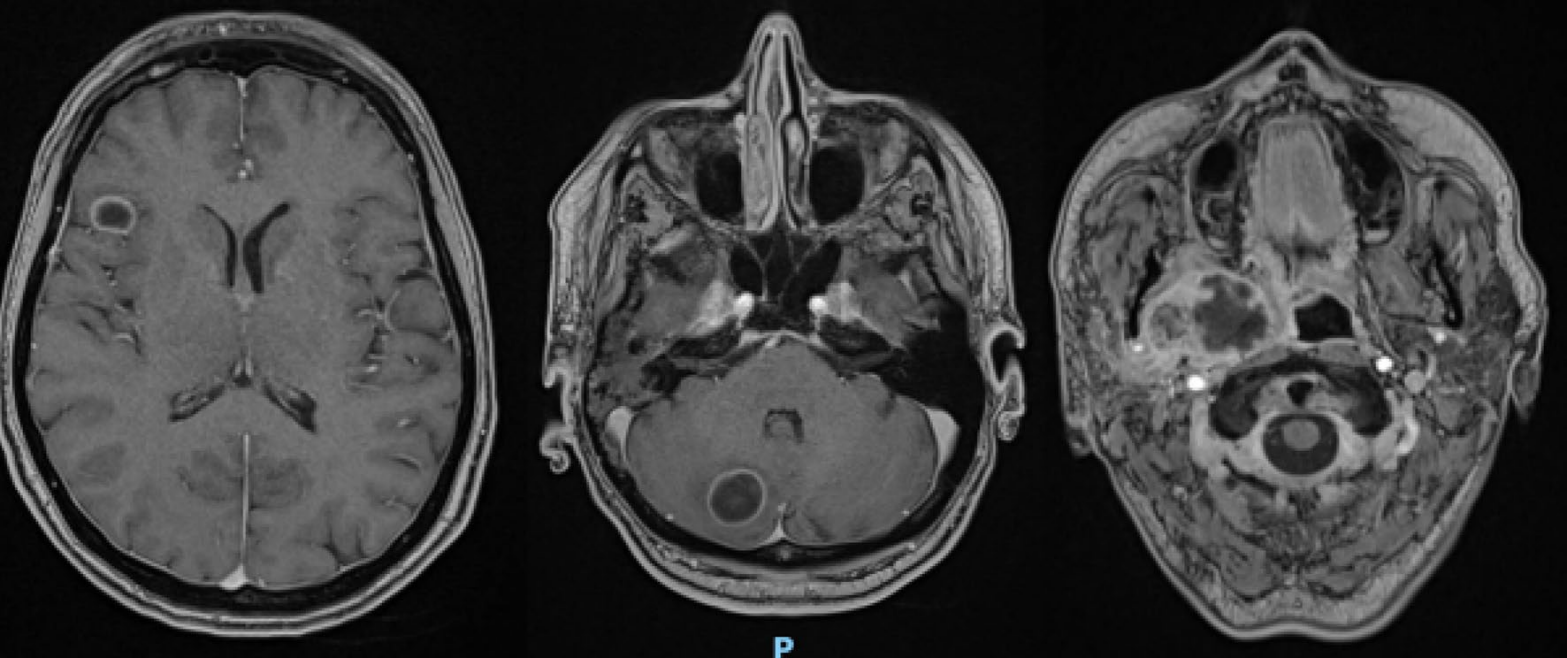
Brain MRI with transverse scans (T1_vibe_dixon sequencies). The presence of a pathological formation in the right lateral pharynx with a mass effect on the airways, with a non-vascularized internal portion, was confirmed; the two lesions at the encephalic and cerebellar level were confirmed, with the diagnosis of neoplastic lesions

**Fig. 6:**
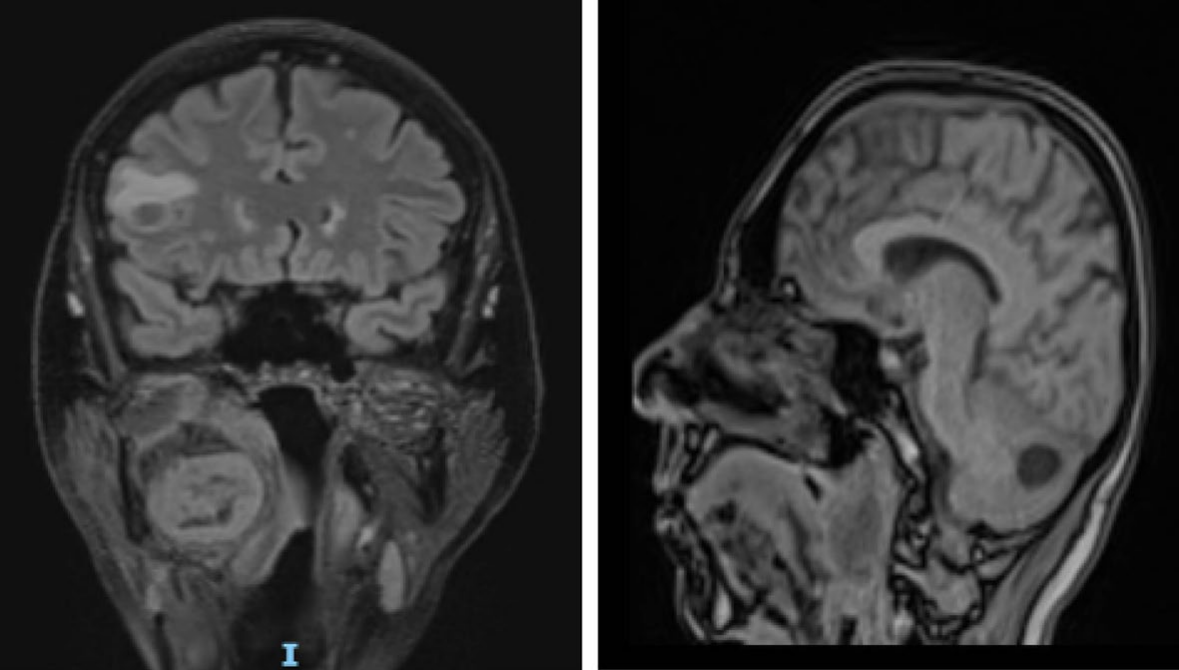
Brain MRI with sagittal section (T2_space 3D dark fluid sequences). Detail on the parapharyngeal formation with evident mass effect on the trachea; detail on the cerebellar formation

Furthermore, brain MRI confirmed the hypothesis of repetitive/neoplastic encephalic lesions rather than abscess ones (Figs. 5 and 6). Antibiotic therapy was progressively decreased until discontinuation.

**Fig. 7:**
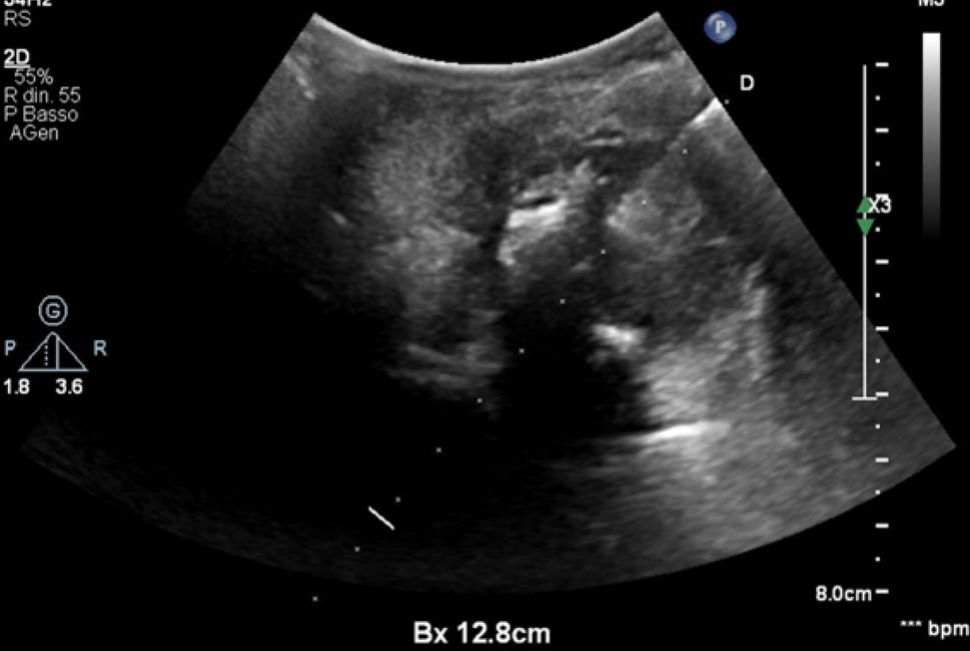
Ultrasound-guided needle biopsy on the mass in the right lateral pharynx; procedure performed with a 20 G needle

An ultrasound guided biopsy was performed by a transcutaneous biopsy with a high median approach at neck level, to characterize the mass in the right tonsillar region (Fig. 7). The histological examination detected myxo-chondroid material incorporating cells with nuclear atypia, indicative for malignant neoplasm (PD-L1 negative; p16+); the final histological diagnosis was sarcomatoid carcinoma.

A subsequent total body CT scan showed bilateral pulmonary nodularities, ilo-mediastinal adenopathic involvement and a suspected lesion of the liver in the VII segment.

At the oncology consult it was decided on a cycle of chemotherapy with cisplatin + 5-fluorouracil + cetuximab scheme; moreover, evaluation was carried out for radiotherapy treatment at the encephalic level.

## Discussion

Masses in the parapharyngeal area are rare and often due to infectious phenomena arising from the oral cavity or pharynx which lead to abscess formation [10]. Less frequently, as in our case, the lesion is neoplastic. Tumours of the parapharyngeal space are rare, accounting for less than 1% of all head and neck neoplasms [11, 12]. Parapharyngeal masses can represent a relevant differential diagnostic challenge for clinicians because of the variety of pathological entities that arise in the parapharyngeal space, and the anatomical and functional complexity of the region [12].

Sparano et al. described a previous case report on a large malignant rhabdoid tumor of the parapharyngeal space with extension to the infratemporal fossa and skull base in 2-year-old girl who had presented with a cervical mass and ipsilateral Horner syndrome [13]. Ide et al. reported the case of sarcomatoid variant of salivary duct carcinoma in a 45-year-old woman, presenting as a 1.5-cm polypoid mass protruded from the retromolar area and focally extended into the pharyngeal wall [14].

Biopsy, fine needle biopsy or typically FNA cytology, plays an essential role in establishing a preoperative diagnosis. Fine-needle aspiration is characterized by a relatively high accuracy in differentiating between benign and malignant masses [15, 16]. The diagnostic accuracy of FNA in subjects affected by PPS masses is relatively low due to hemorrhaging, inadequate specimen collection and other technical problems [5, 17]. Moreover, the seeding of tumor cells should be taken into consideration when performing a transoral biopsy. The rate of non-diagnostic FNA samples could be as high as 25–35% [17, 18]. Tissue sampling could also induce pain, bleeding, and damage to neurovascular structures. Therefore, the use of FNA is controversial [19]. Generally, transoral or percutaneous biopsies should be performed only when the malignant nature of the PPS neoplasm is highly suspected [5]. The FNA procedure is not a first-choice diagnostic tool for benign PPS masses.

Regarding our case, the appearance of a peripheral hyperenhancement with a non-vascularized internal area on both CEUS and CT, evident both at the level of the primary lesion and at the cerebral and cerebellar level, could raise the suspicion of an abscess formation. Otherwise, the clinical presentation was not suggestive of an infectious etiology as there was no fever and the inflammation markers on laboratory tests were within normal limits; furthermore, the patient did not have an immunosuppressive condition.

The completion with the MRI and the biopsy procedure allowed us to reach the specific diagnosis. In our case, it was possible to perform a transcutaneous biopsy with a high median approach at neck level; transoral biopsy procedures from the oral cavity are often reported in the literature [20]. Therefore, it was possible to perform a quick, less painful and less invasive procedure related to the use of ultrasound guidance.

In the literature, almost all of the articles published on biopsy procedures in this setting are case reports. It is interesting to note that there is a debate on which is the best strategy/route both to perform biopsy on the mass and on a surgical point of view. Zhang et a., reported an endoscopic approach to perform ultrasound-guided fine-needle aspiration to diagnose a recurrent nasopharyngeal carcinoma in the parapharyngeal space [21].

In their work, Motta et al. argued that endoscopically assisted transoral approach is a valid technique for treating benign capsulated tumours of the true PPS and some benign capsulated tumours of the superomedial aspect of the carotid space [20].

Galli et al. reviewed 129 consecutive patients submitted to PPS surgery via transcervical-trans-parotid route [22]. Most tumors involved the pre-styloid space (83.7%) [22]. The transcervical-trans-parotid corridor was used in 70.5% of patients, while a pure transcervical route in about a quarter of cases. Early postoperative VII cranial nerve (CN) palsy was present in 32.3% of patients, while X CN deficit in 9.4%. The long-term morbidity rate was 34.1%, with persistent CN impairment detectable in 26.4% of patients; a recurrence occurred in 12 patients (9.4%). In their conclusions, authors argued that the transcervical-transparotid corridor represents the benchmark for surgical management of most of PPS neoplasms [22].

## Conclusions

In cases of masses in the PPS, to perform diagnostic imaging allows a presumptive characterization of the nature of the lesion. A biopsy procedure (core needle or FNA) is indicated in cases in which the mass is suspicious for a malignant lesion or in cases in which strong doubt persists about the nature of the mass. An ultrasound-guided procedure offers clear advantages in terms of reduction in costs, radiation exposure and timing, and is a method capable of accelerating diagnosis. Larger studies in the literature will have to clarify which conditions favor the use of an endoscopic (oral) or transcutaneous approach.
